# Measuring the adequacy of antenatal health care: a national cross-sectional study in Mexico

**DOI:** 10.2471/BLT.15.168302

**Published:** 2016-06-01

**Authors:** Ileana Heredia-Pi, Edson Servan-Mori, Blair G Darney, Hortensia Reyes-Morales, Rafael Lozano

**Affiliations:** a Center for Health System Research, National Institute of Public Health, Av. Universidad #655, 62100, Cuernavaca, Morelos, Mexico.; b Research Department, Federico Gomez Children’s Hospital, Mexico City, Mexico.

## Abstract

**Objective:**

To propose an antenatal care classification for measuring the continuum of health care based on the concept of adequacy: timeliness of entry into antenatal care, number of antenatal care visits and key processes of care.

**Methods:**

In a cross-sectional, retrospective study we used data from the Mexican National Health and Nutrition Survey (ENSANUT) in 2012. This contained self-reported information about antenatal care use by 6494 women during their last pregnancy ending in live birth. Antenatal care was considered to be adequate if a woman attended her first visit during the first trimester of pregnancy, made a minimum of four antenatal care visits and underwent at least seven of the eight recommended procedures during visits. We used multivariate ordinal logistic regression to identify correlates of adequate antenatal care and predicted coverage.

**Findings:**

Based on a population-weighted sample of 9 052 044, 98.4% of women received antenatal care during their last pregnancy, but only 71.5% (95% confidence interval, CI: 69.7 to 73.2) received maternal health care classified as adequate. Significant geographic differences in coverage of care were identified among states. The probability of receiving adequate antenatal care was higher among women of higher socioeconomic status, with more years of schooling and with health insurance.

**Conclusion:**

While basic antenatal care coverage is high in Mexico, adequate care remains low. Efforts by health systems, governments and researchers to measure and improve antenatal care should adopt a more rigorous definition of care to include important elements of quality such as continuity and processes of care.

## Résumé

**Objectif:**

Proposer une classification des soins prénataux afin de mesurer la continuité des soins et leur caractère adéquat: date de début des soins prénataux, nombre de consultations prénatales et principaux processus de soins.

**Méthodes:**

Lors de notre étude rétrospective transversale, nous avons utilisé les données de l'enquête nationale sur la santé et la nutrition (ENSANUT) réalisée au Mexique en 2012. Celle-ci contenait des informations sur le recours aux soins prénataux déclaré par 6494 femmes lors de leur dernière grossesse ayant abouti à une naissance vivante. Les soins prénataux ont été considérés adéquats lorsqu'une femme avait eu sa première consultation au cours du premier trimestre de grossesse, s'était rendue au minimum à quatre consultations prénatales et avait bénéficié d'au moins sept des huit procédures recommandées lors des consultations. Nous avons utilisé une régression logistique ordinale multivariée pour identifier les corrélations entre soins prénataux adéquats et prévisions de la couverture de soins.

**Résultats:**

Sur un échantillon pondéré en fonction de la population de 9 052 044 femmes, 98,4% avaient reçu des soins prénataux lors de leur dernière grossesse, mais seulement 71,5% (intervalle de confiance de 95%: 69,7 à 73,2) avaient reçu des soins jugés adéquats. D'importantes différences géographiques ont été observées entre les États au niveau de la couverture de soins. La probabilité de bénéficier de soins prénataux adéquats était plus forte pour les femmes au statut socioéconomique plus élevé, celles ayant eu une scolarité plus longue et celles disposant d'une assurance maladie.

**Conclusion:**

Au Mexique, si la couverture en matière de soins prénataux de base est élevée, les soins adéquats restent limités. Les systèmes de santé, les gouvernements et les chercheurs, dans leurs efforts pour mesurer et améliorer les soins prénataux, devraient adopter une définition plus rigoureuse de ce type de soins afin d'y inclure d'importants aspects qualitatifs comme la continuité et les processus de soins.

## Resumen

**Objetivo:**

Proponer una clasificación de atención prenatal para medir la continuidad de la atención sanitaria según su idoneidad: momento en que se empieza a recibir atención prenatal, número de visitas de atención prenatal y procesos básicos de atención.

**Métodos:**

En un estudio transversal y retrospectivo se utilizaron datos de la Encuesta Nacional de Salud y Nutrición (ENSANUT) realizada en México en 2012, que contenía información autodeclarada acerca del uso de atención prenatal de 6 494 mujeres durante su último embarazo con nacidos vivos. Se consideró que la atención prenatal era adecuada si una mujer realizaba su primera visita durante el primer trimestre de embarazo, hacía al menos cuatro visitas de atención prenatal y recibía al menos siete de los ocho procedimientos recomendados durante las visitas. Se utilizó una regresión logística ordinal multivariable para identificar las correlaciones de la atención prenatal adecuada y la cobertura prevista.

**Resultados:**

Según una muestra ponderada de la población de 9 052 044 personas, el 98,4% de las mujeres recibieron atención prenatal durante su último embarazo, pero únicamente el 71,5% (intervalo de confianza del 95%: 69,7 a 73,2) recibieron atención sanitaria prenatal clasificada como adecuada. Se identificaron importantes diferencias geográficas en la cobertura de la atención sanitaria entre los estados. La probabilidad de recibir una atención prenatal adecuada era mayor entre mujeres con una mejor situación socioeconómica, más años de escolarización y seguro médico.

**Conclusión:**

Aunque la cobertura básica de atención prenatal es alta en México, su idoneidad sigue siendo escasa. Los esfuerzos realizados por sistemas sanitarios, gobiernos e investigadores para medir y mejorar la atención prenatal deberían adoptar una definición más rigurosa de la misma para incluir elementos importantes de calidad, como la continuidad y los procesos de atención.

## ملخص

الغرض اقتراح تصنيف لخدمات الرعاية فيما قبل الولادة من أجل قياس مدى تواصل تقديم الرعاية الصحية بناءً على مفهوم الكفاية:

البدء في تلقي خدمات الرعاية فيما قبل الولادة في الوقت المناسب، وعدد الزيارات التي يتم فيها تقديم الرعاية فيما قبل الولادة، والإجراءات الرئيسية لتقديم الرعاية.

**الطريقة:**

استخدمنا البيانات المستمدة من الدراسة الاستقصائية الوطنية بشأن الصحة والتغذية في المكسيك (ENSANUT) التي تم إجراؤها في عام 2012، وذلك من خلال دراسة بأثر رجعي متعددة القطاعات. وقد تضمنت البيانات معلومات وردت عن أصحابها بشأن استفادة عدد من النساء يبلغ 6494 امرأة من خدمات الرعاية فيما قبل الولادة أثناء آخر فترة حمل مررن بها أثمرت عن مولود على قيد الحياة. وتم اعتبار الرعاية فيما قبل الولادة رعايةً كافية إذا ما قامت المرأة بالزيارة الأولى لها في الثلاثة أشهر الأولى من الحمل، ثم قامت بأربع زيارات كحد أدنى لتلقي الرعاية فيما قبل الولادة، مع الخضوع لسبعة إجراءات على الأقل من الإجراءات الثمانية الموصى بها أثناء تلك الزيارات. واستخدمنا التحوف اللوجيستي الترتيبي متعدد المتغيرات لتحديد العوامل المرتبطة فيما بينها والمتعلقة بالرعاية الكافية فيما قبل الولادة والتغطية المتوقعة لهذه الرعاية.

**النتائج:**

وفقًا لما تم التوصل إليه من خلال عينة مرجحّة بعدد السكان يبلغ عدد أفرادها 9,052,044، تلقت ‏98.4% من النساء الرعاية فيما قبل الولادة خلال آخر فترة حمل مررن بها، إلا أن ‏71.5% فقط (بنسبة أرجحية مقدارها 95‏%: من 69.7 إلى 73.2) من الرعاية الصحية المقدمة للأمهات تم تصنيفها كرعاية كافية. وتم تحديد بعض الفروق الجغرافية الهامة في التغطية المتعلقة بخدمات الرعاية بين الولايات. وزاد احتمال تلقي الرعاية الكافية فيما قبل الولادة بين النساء اللاتي تتمتعن بمستوى أعلى من الوضع الاجتماعي الاقتصادي، واللاتي تلقين التعليم المدرسي على مدى عدد أكبر من السنوات، واللاتي يتوفر لهن التأمين الصحي.

**الاستنتاج:**

رغم ارتفاع مستوى التغطية المتعلقة بتقديم الرعاية الأساسية فيما قبل الولادة في المكسيك، ظلت الرعاية الكافية منخفضة المستوى. ويجب أن يشمل الجهد المبذول من جانب الجهات المسؤولة عن الأنظمة الصحية، والحكومات، والباحثين لقياس خدمات الرعاية فيما قبل الولادة والعمل على تحسينها تطبيقًا لمفهوم يتناول خدمات الرعاية بأسلوب أكثر دقة بما يشمل العناصر الهامة المتعلقة بمستوى الجودة مثل الاستمرارية والإجراءات المتبعة لتقديم الرعاية.

## 摘要

**目的:**

旨在基于充分性的概念提出一种衡量保健持续性的产前保健分类依据：产前保健时效性、接受产前检查的次数和关键的保健流程。

**方法:**

在一项横断面回溯式研究中，我们采用了 2012 年墨西哥国家健康和营养调查 (ENSANUT) 数据。其中包含 6494 名顺利生产的女性自行报告的信息，这些信息均与她们在怀孕期间的产前保健有关。如果孕妇在怀孕的前三个月进行第一次检查，孕期至少进行四次产前检查，并且在检查过程中至少进行八项推荐程序中的七项程序，那么我们认为该产前保健是充分的。我们采用了多元有序逻辑回归确定充分产前保健和预测覆盖率之间的相关性。

**结果:**

基于 9 052 044 份人口加权样本，98.4% 的孕妇在其上次怀孕期间接受过产前保健，但是仅 71.5% （95% 置信区间：接受的产前保健可归为充分保健。已确定各州之间在保健覆盖率方面存在显著的地区差异。社会经济地位更高、接受教育年限更长且享有医疗保险的女性，接受充分产前保健的可能性更高。

**结论:**

尽管墨西哥地区基本产前保健覆盖率很高，但是充分保健覆盖率却依然很低。医疗系统、政府和调查机构应努力采取一种更为严格的保健定义以将重要的质量因素（例如持续性和保健流程）包括在内，进而衡量和改进产前保健。

## Резюме

**Цель:**

Предложить классификацию дородовой медицинской помощи для оценки процесса непрерывного оказания помощи, в основу которой заложена концепция адекватности. Адекватность определяется своевременностью начала оказания дородовой медицинской помощи, количеством посещений в рамках дородовой помощи и основными процедурами, составляющими медицинскую помощь.

**Методы:**

В ходе одномоментного поперечного ретроспективного исследования были использованы данные Мексиканского национального исследования в области здравоохранения и питания (ENSANUT) 2012 года. Эти данные содержали сообщения 6494 женщин о личном опыте получения дородовой медицинской помощи в течение их последней беременности, закончившейся рождением живого ребенка. Дородовая медицинская помощь считалась адекватной, если женщина впервые посетила учреждение дородовой помощи в течение первого триместра беременности, совершила как минимум четыре таких визита и в ходе этих визитов была подвергнута по меньшей мере семи из восьми рекомендованных процедур. Взаимосвязь между адекватной дородовой медицинской помощью и прогнозируемым охватом была определена с помощью мультиномиальной порядковой логистической регрессии.

**Результаты:**

Из 9 052 044 человек, включенных во взвешенную выборку из генеральной совокупности, 98,4% женщин получали дородовую помощь в течение последней беременности, но только для 71,5% (95%-й доверительный интервал: 69,7–73,2) матерей эта медицинская помощь могла быть классифицирована как адекватная. Между штатами были выявлены значительные различия в оказании медико-санитарной помощи. Адекватную дородовую медицинскую помощь с большей вероятностью получали женщины более высокого социально-экономического статуса, более образованные и имеющие медицинскую страховку.

**Вывод:**

Несмотря на высокий охват базовой дородовой медицинской помощью в Мексике, доля адекватной помощи по-прежнему мала. При реализации мер, предпринимаемых участниками систем здравоохранения, должностными лицами и исследователями для оценки и совершенствования дородовой медицинской помощи, следует пользоваться более точным определением помощи, которое включает такие важные элементы качества, как непрерывность оказания помощи и проводимые в ее рамках процедуры.

## Introduction

Optimizing maternal and infant health requires, but is not limited to, the provision of available and accessible health care delivered by skilled health personnel throughout the antenatal period.^1^ Besides offering the interventions recommended by the World Health Organization (WHO), it is essential to guarantee universal coverage of services within a framework of continued care throughout pregnancy.^2^^–^^8^

Any assessment of maternal care needs to be performed within the framework of human rights.^9^ Strategies towards ending preventable maternal mortality are aimed at the achievement of millennium development goals (up to 2015) and now sustainable development goals in the area of maternal mortality. These goals seek to eliminate inequalities in access to health care and to ensure women receive universal coverage of sexual and reproductive health services that are responsive to women’s needs.^10^

Improving maternal and neonatal health outcomes involves the provision and uptake of antenatal services that are timely (first visit during the first three months of pregnancy), sufficient (at least four antenatal visits) and adequate (with appropriate content). Rarely, however, have these conditions been studied together in the context of low- and middle-income countries.^11^ The majority of studies including these indicators have measured coverage independently – thus reporting high average levels – but have failed to reflect the individual dimension of services provided (women who received comprehensive care and coverage in all indicators).^12^^–^^14^

Efforts to develop indicators to measure the adequacy of antenatal care and the continuum of care throughout the lifecycle^5^ have been continuing for over four decades. Among these, an index to measure the timeliness of the initial antenatal care intervention was proposed in 1973.^15^ However, it overlooked content, thereby ruling out the possibility of evaluating antenatal care through process measures. Impractical for assessing the clinical relevance of care, it has been classified as an indicator of service use only.^15^ Other authors have proposed combining several antenatal health-care indicators,^16^^–^^20^ but have as yet been unable to offer a fully comprehensive solution. In omitting components of the content of care, such indices measure the use of services and not the processes of care that are a necessary condition for evaluating adequacy. Since 2006^21^ numerous studies have been published which include the procedures implemented during antenatal visits. Complex indicators, combining the content of visits with other variables across the continuum of care,^5^ have been developed.^13^^,^^22^ Again, however, none have as yet achieved a fully comprehensive approach to the measurement of antenatal care continuity and adequacy. Some studies have even used local indicators, thus limiting international comparisons, while others have overlooked the usefulness of measuring conditional rather than independent probabilities: that is, measuring the coverage of an indicator conditional on the coverage of another one.^13^^,^^14^^,^^21^^,^^22^

Based on our previous research,^12^ and drawing on population-based data from the most recent health and nutrition survey in Mexico, we propose an antenatal care classification that allows the continuum of services to be measured according to four dimensions of the health care process: access to care delivered by skilled health personnel that is timely, sufficient and with appropriate content. In particular, this study aimed to describe the adequacy of antenatal care for women in the context of the population and geography of Mexico. Using our conditional classification we also aimed to identify the individual factors associated with the type of antenatal care received by women during their most recent pregnancy, at both the household and community levels.

## Methods

### Study design and data source

We report a retrospective analysis of data from the Mexican National Health and Nutrition Survey done in 2012 (Spanish acronym: ENSANUT). This was a cross-sectional, population-based household survey, based on a national population of 115 170 278, with sampling representative at the state level (Mexico has 32 states) and by rural/urban stratum. The survey was designed to estimate the prevalence and proportions of health and nutrition conditions, access to services, health determinants, as well as coverage of health-care services for specific and distinct groups of the Mexican population.^23^ Survey data (available to the general public^24^) were collected in a single interview after obtaining the informed consent of each participant and the approval of the ethics, research and biosecurity committees of the National Institute of Public Health in Mexico.

We used data from the survey’s reproductive health module, which had been applied to a random subsample of 23 056 women aged 12–49 years. From these, we selected women who had delivered their last live birth from 2006 onwards and who had been asked a series of questions about their use of antenatal care and obstetric services. We excluded those who had provided incomplete information on the relevant variables. A comparison of the sociodemographic and health-related characteristics of women who did and did not participate in the analytical sample yielded no statistically significant differences.

The dependent variables, i.e. the four dimensions of continuity and adequacy of antenatal care, were: (i) skilled health care (antenatal care provided by a nurse or a physician); (ii) timely (initial antenatal care visit during the first trimester of pregnancy); (iii) sufficient (at least four antenatal care visits during the pregnancy); and (iv) appropriate in content (an indicator summarizing the procedures and processes of care provided during antenatal care).

For the indicator of appropriate content we selected eight of the 12 procedure items used in the survey: weight; height; blood pressure; general urine analysis; blood analysis; tetanus vaccination; prescription of folic acid; and prescription of vitamins iron or dietary supplements. We excluded human immunodeficiency virus testing (since the official guidelines are that this test should be applied only to high-risk women^25^ and we were unable to ascertain this information); ultrasound examination (because this is not considered a required procedure by the authorities in Mexico^25^ and because of the inconsistencies in scientific evidence regarding its importance); and glucose and syphilis testing (because these tests were grouped together in the survey item and we could not distinguish between them). The previous literature has not been able to identify a single cut-off to classify antenatal care content as adequate or not; studies have considered cut-offs from 60% to 80% of the total of procedures measured.^14^^,^^21^^,^^22^^,^^26^^–^^28^ We classified women in the highest quintile of received procedures as having received an adequate content of care (appropriate in content). This corresponded to seven out of eight of the procedure items received. In line with previous methods,^12^ all interventions or procedures provided during antenatal care visits were weighted equally.

We divided the study sample into three outcome categories: received adequate antenatal care (delivered by skilled health personnel, timely, sufficient and with appropriate content); received inadequate antenatal care (services which did not fully comply with these criteria); or received no antenatal care from a health facility.

### Covariates

We included individual and household-level covariates. At the individual level we recorded data on the women’s sociodemographic characteristics and utilization of health-care services (antenatal and obstetric care). These were: woman’s age (12–19, 20–29 or 30–49 years at the time of her last live birth), education (0, 1–6, 7–9, 10–12 or ≥ 13 years of schooling completed), previous parity (0, 1 or ≥ 2 live births), history of infant death (stillbirth or death within the first year of life), history of miscarriage or induced abortion (we were unable to distinguish between spontaneous and induced abortion), and year of the index live birth (2006–2007, 2008–2009 or 2010–2012). Type of health insurance was classified as: none, Social Security, or *Seguro Popular de Salud* (an employment-based health insurance for people working in the informal sector or without other access to insurance); women with private health insurance were excluded as they were a very small percentage. In addition to our definition of adequacy described above, we classified the type of health facility where the majority of antenatal care was received as: social security, ministry of health, private or other (midwife or home). We included six binary indicators (scored yes/no) for diagnosis of a health problem during pregnancy (high blood pressure, vaginal bleeding, threat of miscarriage, pre-eclampsia or eclampsia, gestational diabetes or infections).

At the household level, we created binary (yes/no) indicators for indigenous status (a household in which the head of the family, a spouse or an older relative self-identifies as indigenous or speaks an indigenous language^29^), and whether the household was a beneficiary from the *Oportunidades* social programme (now called *Prospera*). We included an asset and housing index as a measure of socioeconomic status based on assets and household infrastructure, developed using polychoric correlation matrices (range: −5.9 to 1.8),^30^^,^^31^ and collapsed into terciles (low, middle or high), whereby higher values denoted a greater number of assets and better housing conditions. We also included an indicator for the location of the household, based on community and state-level indicators and population: rural (< 2500 residents), urban (2500–100 000 residents) or metropolitan (> 100 000 residents). Finally, we included the level of marginalization (low or high), which is a community-level index based on lack of access to education, inadequate housing and perceived insufficient income.^32^

### Analysis

The data were analysed using the Stata package version 13.2 (StataCorp LP, College Station, United States of America). First, we estimated the consecutive independent and conditional probabilities for each dimension of antenatal care. Independent coverage was the percentage of the population receiving an intervention, measuring the coverage of each indicator separately. Conditional coverage refers to full compliance with antenatal care indicators, measuring the coverage of each indicator conditioned on the coverage of the previous one. The socioeconomic, demographic and health profiles of the women surveyed were then characterized by type of antenatal care received (adequate, inadequate or none). We then estimated adequate antenatal care coverage in the different states of Mexico. Finally, we produced population estimates for all results by the individual sampling weights and accounting for the complex survey design.

To identify the key sociodemographic factors associated with the antenatal care services used, we next used an ordinal logistic regression model^33^ for the categorical outcome (none = 0, inadequate = 1, adequate = 2). All covariates previously mentioned were included in this model, except diagnosis of a health condition during pregnancy, because this is a time-dependent confounder that can be an effect of adequate antenatal care as well as a cause of more frequent subsequent antenatal care.

For ease of interpretation we calculated marginal effect probabilities and the corresponding 95% confidence intervals (CI). Marginal effects are multivariables predicted for each category of the outcome, holding all other covariates at their median levels. Marginal effects measure discrete change for binary independent variables and measure the instantaneous rate of change for continuous variables. These analyses were implemented using the *mfx* command in Stata.

## Results

We selected 7206 women and after excluding 712 (9.8%) respondents with incomplete data, the sample for analysis was 6494 (90.1%) women (population-weighted sample: 9 052 044). Of these women, 4630 received adequate antenatal care, 1718 inadequate antenatal care and 146 reported having no antenatal care. Based on population-weighted numbers, the independent analysis of the probabilities of coverage estimated that 98.4% of women received antenatal care by skilled health personnel, 83.2% received care that was timely, 91.4% care that was sufficient and 84.7% received care with the appropriate number of antenatal care processes (Table 1). However, the conditional analysis showed that only 71.5% women (95% CI: 69.7 to 73.2) with access to services delivered by skilled health personnel received adequate antenatal care (population-weighted number: 6 470 401 women); 1.6% (95% CI: 1.2 to 2.0) received no antenatal care (population-weighted number: 2 439 526) and 27.0% (95% CI: 25.3 to 28.7) received inadequate antenatal care (population-weighted number: 142 117).

**Table 1:** Independent and conditional analyses of the coverage of the dimensions of antenatal care among pregnant women in a national retrospective study, Mexico, 2012

Dimension of antenatal care^a^	% (95% CI)	
	Independent coverage	Conditional coverage
Skilled	98.4 (98.1 to 98.8)	98.4 (98.1 to 98.8)
Timely	83.2 (81.8 to 84.6)	83.2 (81.8 to 84.6)
Sufficient	91.4 (90.3 to 92.5)	79.9 (78.4 to 81.4)
Appropriate	84.7 (83.3 to 86.2)	71.5 (69.7 to 73.2)

CI: confidence interval.

^a^ Skilled (antenatal care provided by a nurse or a physician); timely (initial antenatal care visit during first trimester of pregnancy); sufficient (≥ 4 antenatal care visits during pregnancy); appropriate (visits included at least 7/8 of recommended basic care procedures: measurement of height, weight, and blood pressure, urine analysis, blood examination, tetanus vaccine, and prescription of folic acid as well as vitamin/iron/food supplements.

Note: Sample *n* = 6494; sample weighted to population *n* = 9 052 044. Independent coverage was the percentage of the population receiving an intervention, measuring the coverage of each indicator separately. Conditional coverage refers to full compliance with antenatal care indicators, measuring the coverage of each indicator conditional on the coverage of the previous one.

**Fig. 1:**
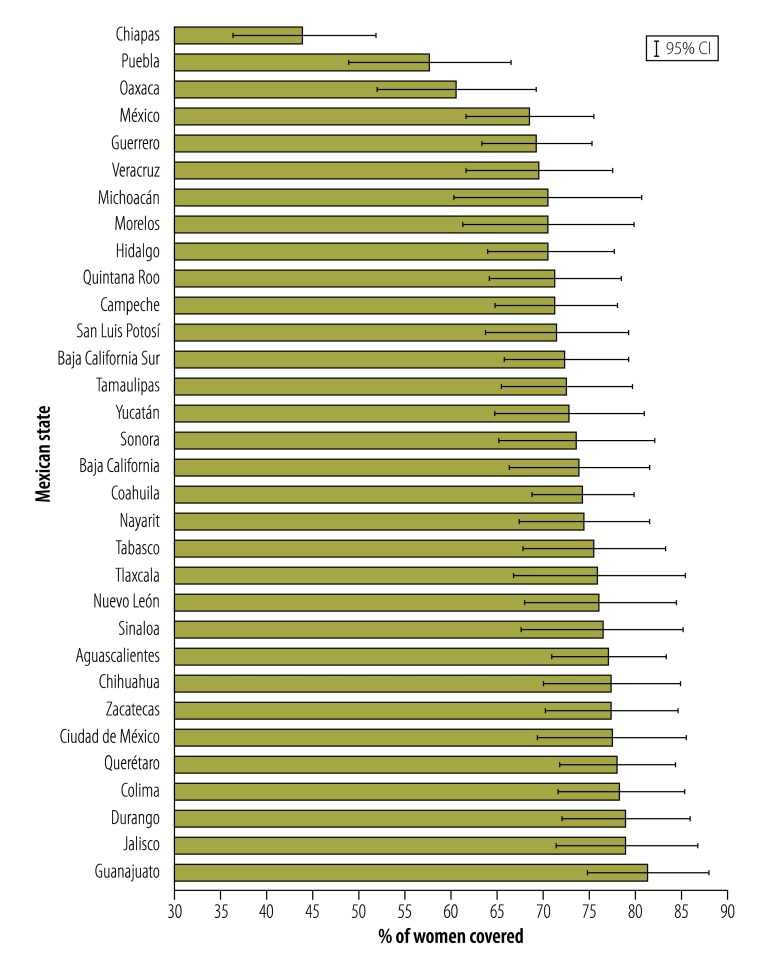
Percentage of women by state with adequate antenatal care in a national retrospective study, Mexico, 2012

Fig. 1 shows the crude levels of adequate antenatal care coverage in the 32 Mexican states. Three states had very low coverage levels: Chiapas (44.2%), Puebla (57.9%) and Oaxaca (60.8%). The coverage in these states was significantly lower (non-overlapping CI, *P* < 0.001) compared with the six states with the highest coverage: Guanajuato (81.6%), Jalisco (79.6%), Durango (79.2%), Colima (78.7%), Querétaro (78.3%) and Mexico City (77.7%).

CI: confidence interval.

Note: Data values are shown for the three highest and six lowest states (*P* < 0.001). Adequate antenatal care was skilled (provided by a nurse or a physician); timely (initial visit during first trimester of pregnancy); sufficient (≥ 4 visits during pregnancy); and appropriate (visits included at least 7/8 of recommended basic care procedures).

When comparing across the three groups (no antenatal care, inadequate care and adequate care), we observed overall socioeconomic disparities. Women who received antenatal care had had more years of schooling, were older and had fewer children at the time of their last delivery (*P* < 0.001; Table 2). A smaller percentage of women receiving antenatal care had experienced previous stillbirths, were from indigenous families and were benefiting from the *Oportunidades* social programme (*P* < 0.001). Women who received antenatal care lived primarily in households with more assets and better housing conditions, located in less marginalized metropolitan areas (all *P* < 0.001).

**Table 2:** Individual and household characteristics of women by access to and adequacy of antenatal care in a national retrospective study, Mexico, 2012

Characteristic	% (95% CI)			*P*^b^
	No antenatal care	Inadequate antenatal care	Adequate antenatal care^a^	
**Individual **				
No. of years in school				
0	22.3 (13.6 to 34.3)	6.4 (5.0 to 8.1)	3.2 (2.4 to 4.4)	< 0.001
1–6	34.3 (24.4 to 45.8)	25.1 (22.1 to 28.3)	20.3 (18.5 to 22.3)	
7–9	31.5 (22.3 to 42.6)	41.6 (38.0 to 45.3)	36.8 (34.4 to 39.2)	
10–12	10.1 (3.8 to 24.2)	20.2 (17.3 to 23.4)	26.4 (24.2 to 28.8)	
≥ 13	1.7 (0.4 to 7.8)	6.7 (5.0 to 9.0)	13.2 (11.6 to 15.1)	
Age at time of last delivery, years				
12–19	22.6 (15.0 to 32.5)	25.3 (22.2 to 28.7)	18.0 (16.4 to 19.9)	< 0.001
20–29	48.6 (37.7 to 59.7)	51.0 (47.5 to 54.6)	54.5 (52.3 to 56.7)	
30–49	28.8 (20.6 to 38.7)	23.6 (20.9 to 26.7)	27.4 (25.6 to 29.4)	
No. of children at the time of last delivery				
0	35.1 (23.4 to 48.8)	35.5 (31.8 to 39.5)	31.3 (29.3 to 33.5)	0.001
1	22.3 (14.9 to 32.1)	27.3 (24.1 to 30.7)	33.9 (31.8 to 36.0)	
≥ 2	42.6 (31.5 to 54.5)	37.2 (33.5 to 41.1)	34.8 (32.7 to 36.9)	
Year of obstetric episode				
2006–2007	23.6 (16.1 to 33.2)	28.3 (25.0 to 31.8)	26.8 (25.0 to 28.8)	< 0.05
2008–2009	41.8 (31.0 to 53.5)	32.0 (28.5 to 35.6)	38.6 (36.3 to 41.0)	
2010–2012	34.6 (25.4 to 45.1)	39.7 (36.2 to 43.3)	34.6 (32.3 to 36.8)	
Infant death (stillbirth or death within the first year of life)	13.8 (7.1 to 24.9)	3.6 (2.7 to 4.8)	3.9 (3.1 to 4.8)	< 0.001
At least one miscarriage or abortion	20.5 (12.9 to 31.0)	13.1 (10.9 to 15.6)	15.1 (13.7 to 16.6)	0.138
Health insurance				
Social security	12.1 (6.0 to 23.1)	19.9 (16.9 to 23.2)	34.2 (31.9 to 36.6)	< 0.01
*Seguro popular de salud*	57.7 (46.4 to 68.2)	52.0 (48.0 to 55.9)	44.5 (42.2 to 46.8)	
None	30.2 (21.7 to 40.3)	28.2 (24.6 to 32.0)	21.3 (19.0 to 23.8)	
Frequent antenatal care provider				
Social security	NA	21.1 (18.2 to 24.2)	32.2 (29.9 to 34.6)	< 0.001
Ministry of health	NA	52.2 (48.5 to 56.0)	42.7 (40.1 to 45.4)	
Private	NA	23.5 (20.2 to 27.1)	22.8 (20.7 to 25.1)	
Other	NA	3.2 (2.3 to 4.4)	2.2 (1.7 to 2.9)	
Health problem diagnosed during pregnancy^c^	NA	55.2 (51.3 to 59.1)	60.4 (58.0 to 62.7)	0.027
**Household **				
Indigenous	43.8 (31.9 to 56.5)	12.1 (10.1 to 14.4)	7.9 (6.7 to 9.4)	< 0.001
*Oportunidades* beneficiary	41.4 (30.1 to 53.6)	26.8 (23.7 to 30.1)	20.9 (19.2 to 22.7)	< 0.001
Asset and housing index (tercile)				
Low	72.4 (60.5 to 81.8)	42.5 (38.6 to 46.4)	29.9 (27.9 to 32.1)	< 0.001
Middle	17.8 (11.5 to 26.7)	33.4 (29.6 to 37.4)	32.8 (30.7 to 35.0)	
High	9.7 (3.7 to 23.4)	24.1 (20.9 to 27.8)	37.2 (34.7 to 39.8)	
Area of residence				
Rural	47.9 (36.2 to 59.8)	25.5 (22.6 to 28.6)	21.6 (20.0 to 23.2)	< 0.001
Urban	15.4 (9.6 to 23.8)	23.3 (20.3 to 26.6)	19.2 (17.8 to 20.6)	
Metropolitan	36.7 (25.2 to 49.9)	51.2 (47.3 to 55.2)	59.3 (57.2 to 61.4)	
Marginalization index				
Low	56.5 (44.6 to 67.6)	72.7 (69.4 to 75.7)	77.6 (76.0 to 79.1)	< 0.001
High	43.5 (32.4 to 55.4)	27.3 (24.3 to 30.6)	22.4 (20.9 to 24.0)	

CI: confidence interval; NA: data not applicable.

^a^ Adequate: antenatal care that was skilled (provided by a nurse or a physician); timely (initial visit during first trimester of pregnancy); sufficient (≥ 4 visits during pregnancy); and appropriate (visits included at least 7/8 of recommended basic care procedures).

^b^ *P*-values refer to the test of equality or similar distributions across the three groups; values below 0.05 signify that distributions were statistically different with 95% confidence. Estimates included the effect of the survey design.

^c^ Problems included high blood pressure, vaginal bleeding, threat of miscarriage, pre-eclampsia or eclampsia, gestational diabetes or infections.

Notes: No antenatal care: sample *n* = 146; weighted sample *n* = 142 117. Inadequate antenatal care: sample *n* = 1718; weighted sample *n* = 2 439 526. Adequate antenatal care: sample *n* = 4630; weighted sample *n* = 6 470 401.

The results of the multivariate ordered logit model confirmed the bivariate analyses (Table 3). The covariates most highly correlated with receipt of adequate antenatal care were mother’s education, health insurance, indigenous status and household wealth (all *P* < 0.001). For women with ≥ 13 years of education the probability of having adequate antenatal care was 28.2 percentage-points (95% CI: 15.3 to 41.0) higher compared with those with no education. The probability of having adequate antenatal care was 15.7 percentage points (95% CI: 9.2 to 22.3) higher for women having health insurance via social security and was 6.9 percentage points (95% CI: 1.3 to 12.5) higher for those with *Seguro Popular de Salud* compared with women without health insurance. Adequate antenatal care was 8.3 percentage points (95% CI: –14.2 to –2.4) lower among indigenous women than non-indigenous women, and was 13.1 percentage points (95% CI: 6.5 to 19.7) higher among women in the highest tertile of socioeconomic status compared with those in the lowest socioeconomic status tertile. The type of facility where most antenatal care was received was not a statistically significant variable and was therefore deleted from the models).

**Table 3:** Ordered logit model of access to and adequacy of antenatal care among women in a national retrospective study, Mexico, 2012

Characteristic	Marginal effects % (95% CI)^a^		
	No antenatal care	Inadequate antenatal care	Adequate antenatal care^b^
**Individual **			
No. of years in school			
0	Ref	Ref	Ref
1–6	−2.7 (–5.0 to –0.4)	−13.0 (–20.8 to –5.2)	15.6 (5.9 to 25.4)
7–9	−2.7 (–5.1 to –0.3)	−12.9 (–20.8 to –5.0)	15.6 (5.6 to 25.6)
10–12	−3.5 (–6.0 to –1.0)	−19.9 (–28.5 to –11.2)	23.4 (12.7 to 34.0)
≥ 13	−3.9 (–6.6 to –1.2)	−24.2 (–35.0 to –13.4)	28.2 (15.3 to 41.0)
No. of children at time of last delivery			
0	Ref	Ref	Ref
1	−1.5 (–2.9 to –0.2)	−6.1 (–10.8 to –1.4)	7.6 (1.9 to 13.3)
≥ 2	−0.8 (–2.2 to 0.7)	−2.7 (–7.9 to 2.4)	3.5 (–3.0 to 10.0)
Health insurance			
None	Ref	Ref	Ref
Social security	−2.7 (–4.3 to –1.1)	−13.0 (–18.9 to –7.2)	15.7 (9.2 to 22.3)
*Seguro popular de salud*	−1.4 (–2.7 to –0.1)	−5.5 (–10.1 to –0.8)	6.9 (1.3 to 12.5)
**Household **			
Indigenous			
No	Ref	Ref	Ref
Yes	2.5 (0.1 to 4.9)	5.8 (1.4 to 10.2)	−8.3 (–14.2 to –2.4)
Asset and housing index (tercile)			
Low	Ref	Ref	Ref
Middle	−1.1 (–2.3 to 0.03)	−4.3 (–8.7 to 0.1)	5.4 (0.1 to 10.7)
High	−2.3 (–3.9 to –0.8)	−10.7 (–16.5 to –4.9)	13.1 (6.5 to 19.7)

CI: confidence interval; Ref: reference group.

^a^ Values are marginal effects expressed as percentage points. Estimates included the effect of the survey design. Models were also adjusted by age at the time of last delivery (years), children dead at childbirth or during the first year, at least one miscarriage or abortion, *Oportunidades* beneficiary, the year of obstetric episode, and characteristics of place of residence (shown in Table 2). Covariates were not statistically significant.

^b^ Adequate: antenatal care that was skilled (provided by a nurse or a physician); timely (initial visit during first trimester of pregnancy); sufficient (≥ 4 visits during pregnancy); and appropriate (visits included at least 7/8 of recommended basic care procedures).

Notes: Sample *n* = 6494; weighted sample *n* = 9 052 044. Post-estimation test showed that the regression model was correctly specified: _hat *P* < 0.001, _hatsq *P* = –0.63.

## Discussion

We offer an approach to measuring the adequacy of antenatal care services in Mexico that was skilled, timely, sufficient and appropriate. The study is based on nationally representative data, and we believe it contributes a more comprehensive classification of antenatal care received than those proposed by previous studies.^11^^–^^14^ Only 71.5% of women attended by skilled health personnel in Mexico received adequate antenatal care during their last pregnancy and the probabilities of receiving adequate care were higher among women with more years of schooling, health insurance and higher socioeconomic status.

Previous efforts to evaluate prenatal care through indicators of antenatal care have not considered the content of care,^15^^–^^20^ but only the opportunity and/or frequency of care. Additionally, those studies were based on estimating coverage of each indicator separately or considering different thresholds for each of the components (for example, the number of antenatal care visits recommended), which represents a barrier for international comparisons. Our approach is more comprehensive and patient-centred and combines all the indicators we used into a measure that is internationally comparable and that allows the identification of women who receive timely, frequent, sufficient and appropriate care. This approach focuses on continuity, process and context of care, instead of more narrowly on access and frequency of care. By identifying women who receive each type of antenatal care, this measure can be used to identify which specific components of the antenatal process are not being received, and to show gaps in coverage among population groups. The approach allows the adequacy of antenatal care to be monitored globally, nationally and at the health facility level. To achieve better maternal and neonatal health outcomes, decision-makers and policy developers can use this relatively simple approach as a proxy for the performance of antenatal care programmes and identify gaps in adequacy of antenatal care services.^22^

Our estimates of the likelihood of receiving antenatal care from skilled providers and evaluations of the adequacy of care show that inequities persist in Mexico, with both indicators more likely to be met for women of higher socioeconomic status. Our results revealed that 1.6% of women (142 117 at the population level) reported having no antenatal care at all during their last pregnancy. Most of these women had none or few years of schooling, low socioeconomic status and no health insurance. They also belonged to indigenous households and resided in highly marginalized rural areas. It was not possible to evaluate the continuity and adequacy of care for these women; we were only able to identify the gap that persists in antenatal care access among certain population groups: those who are the most vulnerable. Our findings are consistent with those of other studies which indicated coverage gaps among specific populations and demonstrated that pregnant women younger than 25 years, who had fewer years of schooling, resided in rural areas and belonged to households and communities with low socioeconomic status, were at greater risk of receiving inadequate antenatal care.^12^^,^^14^^,^^22^ Similarly, our results are congruent with a study from Zambia showing gaps in the continuity and adequacy of care received by pregnant women, with a very low percentage of these women receiving adequate antenatal care.^26^

There are several limitations to our proposed comprehensive indicator of quality of antenatal care. Clearly, the prerequisites for providing women with quality services are that antenatal care is available and is accessible. However, supplies and medical teams must also be available at health facilities, together with adequate information systems to ensure a continuum of information on the women’s past events, general background and relevant characteristics.^34^ The present study was unable to evaluate structural elements of health care quality proposed by the Donabedian conceptual framework: structure, processes and outcomes.^35^^,^^36^

We took into account some features related to the supply of antenatal care services, although these were self-reported by the women. Nevertheless, our analysis did not allow us to evaluate the additional quality dimensions of services proposed by other theoretical frameworks, such as technical quality, interpersonal quality and amenities^37^ or efficacy, effectiveness, acceptability, efficiency, environment and empathy.^38^^–^^40^ This highlights the need to follow-up patients to incorporate some of these features into future health surveys and patient administrative registries and to incorporate quality dimensions in future studies.

Another limitation is that the data analysis may have been affected by recall bias regarding the processes of care, because women may have been unable to remember the functions or names of all the processes received and therefore underreported or reported their experiences inadequately. There may also be an effect due to inaccurate weighting of the processes of care: with no literature available on prioritizing the care processes, we chose to weight them all equally.

To validate the proposed metric, future studies in Mexico can consider different approaches. These might include: consulting maternal health-care experts (for example, using Delphi methods^41^) about proposed quality measures and their assessment; a rigorous review of hospital, clinic or other types of administrative records; benchmarking our results against those of countries with a similar demographic, social and economic profile; and comparing our estimates with population surveys that are similar in timing and design.

Our analysis was an attempt to define an effective antenatal care coverage indicator for Mexico by combining effective access to the required health services with other dimensions, specifically the timeliness, sufficiency and appropriate content or procedures of antenatal care. Future studies will need to focus on generating more comprehensive indicators for measuring quality of antenatal care, including patient and provider-centred indicators,^1^^,^^7^ and aligning the information obtained from administrative sources and clinical records with population and patient surveys. Our study has shown that important challenges still prevent Mexican women from receiving antenatal care services that meet WHO recommendations for equity in access and a continuum of maternal care.^7^ To confront these challenges, the Mexican health sector needs to strengthen its response capacity by not only guaranteeing women access to antenatal care, but also ensuring sufficient antenatal care interventions and a high quality in all aspects of care.
